# Supplementary material for: Content-rich biological network constructed by mining PubMed abstracts
Source: BMC Bioinformatics. 2004 Oct 8;5:147. doi: 10.1186/1471-2105-5-147 (PMC528731; doi:10.1186/1471-2105-5-147)
Supplement: Additional File 2 — The original results of the above study (non-essential files are deleted to keep the file size under the limit set by BMC bioinformatics). [file 1471-2105-5-147-S2.bz2 › chilibotAdditionalFile2/dip10/703ID10809785E18036/html/MALF_MALK.html]

 


 **MALF** and **MALK** 
  
Found 40 abstracts in PubMed, retrieved 10.  
 

 What does Google say? 
 PDF only 
| .edu only 

---

**Interactive relationship** (e.g. stimulation, inhibition, etc)

**Neutral relationship**- To this end, single cysteine residues were introduced into each subunit at several positions and the respective  **malF**  and malG alleles were individually co expressed with each of the  **malK**  alleles.  Ref: 10809785 J Biol Chem, 2000
- The maltose transport complex of Escherichia coli, a member of the ATP binding cassette ABC superfamily, is made up of two nucleotide binding subunits,  **MalK**  2, which hydrolyze ATP with positive cooperativity, and two transmembrane subunits,  **MalF**  and MalG.  Ref: 12093921 Proc Natl Acad Sci U S A, 2002
- The results suggest that residues Ala 85, Lys 106, Val 114, and Val 117 in the helical segment of  **MalK** , to different extents, participate in constitution of asymmetric interaction sites with the EAA loops of  **MalF**  and MalG.  Ref: 10809785 J Biol Chem, 2000

**Non-interactive relationship** (e.g. studied together, co-existance, homology, etc.)

- The maltose transporter contains two transmembrane subunits,  **MalF**  and MalG, and two copies of a nucleotide hydrolyzing subunit,  **MalK** .  Ref: 11150310 J Biol Chem, 2001
- This uncoupled activity was not due to dissociation of the  **MalK**  ATPase subunit from the integral membrane protein  **MalF**  and MalG subunits.  Ref: 10648525 J Bacteriol, 2000
- First, we optimized the yield in purified complex protein by taking advantage of a newly constructed expression plasmid that carries the  **malK** ,  **malF**  and malG genes in tandem orientation.  Ref: 12225853 Biochim Biophys Acta, 2002
- ATPase activity was not due to dissociation of the  **MalK**  subunit from the integral membrane proteins  **MalF**  and MalG but could not be further stimulated by trehalose maltose binding protein TMBP, be it the native protein as isolated from T. litoralis or the soluble engineered protein.  Ref: 11453995 Eur J Biochem, 2001
- We have investigated the proximity of residues in a conserved sequence EAA loop of  **MalF**  and MalG to residues in a helical segment of the  **MalK**  subunits by means of site directed chemical cross linking.  Ref: 10809785 J Biol Chem, 2000
- We report the characterization of TrmB, a protein of 3 00 apparent molecular weight, that is involved in the maltose specific regulation of a gene cluster in Thermococcus litoralis, malE  **malF**  malG orf trmB  **malK** , encoding a binding protein dependent ABC transporter for trehalose and maltose.  Ref: 12426307 J Biol Chem, 2003
- Analysis of flanking database sequences from M. tuberculosis and M. leprae revealed the presence of  **malF** , malG and  **malK**  like genes.  Ref: 10817516 Microbios, 2000
- The binding protein dependent maltose transport system of enterobacteria MalFGK 2, a member of the ATP binding cassette ABC transporter superfamily, is composed of two integral membrane proteins,  **MalF**  and MalG, and of two copies of an ATPase subunit,  **MalK** , which hydrolyze ATP, thus energizing the translocation process.  Ref: 10809785 J Biol Chem, 2000
